# Modeling of Causes of Sina Weibo Continuance Intention with Mediation of Gender Effects

**DOI:** 10.3389/fpsyg.2016.00619

**Published:** 2016-04-29

**Authors:** Lingyu Wang, Wenguo Zhao, Xianghong Sun, Rui Zheng, Weina Qu

**Affiliations:** ^1^Institute of Psychology, Chinese Academy of SciencesBeijing, China; ^2^University of Chinese Academy of SciencesBeijing, China; ^3^School of Educational Science, Ludong UniversityYantai, China

**Keywords:** microblogging, Sina Weibo, continuance intention, expectation confirmation model, gender differences

## Abstract

Sina Weibo is a Twitter-like social networking site and one of the most popular microblogging services in China. This study aims to examine the factors that influence the intentions of users to continue using this site. This paper synthesizes the expectation confirmation model, constructs of habit and perceived critical mass, and the gender effect to construct a theoretical model to explain and predict these user intentions. The model is then tested via an online survey of 498 Sina Weibo users and partial least squares (PLS) modeling. The results indicate that the continuance intention of users is directly predicted by their perceived usefulness of the service (β = 0.299), their satisfaction (β = 0.208), and their habits (β = 0.389), which jointly explain 65.9% of the variance in intention. In addition to the effects of these predictors on the continuance intentions of Sina Weibo users, an assessment of the moderating effect of gender suggests that habit plays a more important role for females than for males in continuance intention, but perceived usefulness seems to be more important for males than for females. The implications of these findings are then discussed.

## Introduction

With the rapid development of Internet technology, we have entered the web 2.0 era. Social media and especially social networking sites (SNSs) have become essential parts of personal life. Millions of people worldwide are using SNSs for commenting, sharing photos, and interacting with others. Facebook, MySpace, LinkedIn, and Twitter are examples of popular SNSs. In China, Sina Weibo (“weibo” means “microblog” in Chinese), which is a Twitter-like microblogging service, is one of the most popular SNSs.

As a microblogging platform, Sina Weibo allows users to post short messages that are limited to 140 characters, to comment on each other’s updates, and to share music, videos, and photos. Additionally, users can share their daily activities, interests, and opinions, build new relationships and maintain their existing relationships with family and friends. This is an interesting social activity which allows individuals interact with each and feel connected with a family, a peer group, or a society. According to Self-determination theory (SDT, Ryan and Deci, 2000), the need of relatedness is a basic psychological need, which refers to the need to feel belongingness and connectedness with others (Harlow, 1958; Baumeister and Leary, 1995; Ryan and Deci, 2000). Sina Weibo provides opportunities for satisfying this need. Sina Weibo offers a unique approach to online information diffusion and sharing and provides the opportunity for Chinese citizens to express their opinions about the government and politics, which facilitates civic engagement and potentially impacts the county’s political and social development. Therefore, this site plays an important role in the lives of Chinese individuals.

There are many microblogging services in China, including the two leading ones, Sina Weibo and Tencent Weibo, as well as other platforms such as NetEase, Sohu, and Baidu. However, Sina Weibo is the most influential and popular service. Typically, when an individual mentions Weibo, that person is referring to the Sina microblog. Thus, we chose Sina Weibo as the research platform. This site has attracted millions of users since its launch in China in 2009. As of June 2013, there were approximately 331 million Weibo users, representing 56% of Chinese Internet users (CNNIC, 2013). Notably, the earliest Chinese microblogging services, such as Jiwai and Fanfou, have been shut down. The sharp increase in Sina Weibo usage indicates that it must possess some factors that hold the attention of its users. Thus, we attempted to elucidate the elements that contribute to this continuance intention.

Despite the significance of this SNS, little is known regarding the factors that underlie its public interest. In the short history of Weibo, comprehensive relevant research has not been conducted. Previous studies on Weibo have mainly focused on its basic statistical characteristics (Chen et al., 2011; Guo et al., 2012; Tian and Zheng, 2012), usage patterns (Zhang and Pentina, 2012), user interests (Liu et al., 2012), and microblogging activities in special use cases (Guo and Huang, 2013; Wang et al., 2013).

Of course, there are many studies that focus on the mechanisms of continuance intentions and behaviors in the context of SNSs (Shi et al., 2009; Park and Lee, 2010; Barnes and Bohringer, 2011; Kim, 2011; Yang and Lai, 2011; Al-Debei et al., 2013; Ku et al., 2013; Yeh et al., 2013; Yin et al., 2013). For example, Al-Debei et al. (2013) examined continuance participation intentions and behaviors on Facebook. Additionally, Barnes and Bohringer (2011) modeled the continuance intention for microblogging services based on similar reports involving Twitter. However, Sina Weibo is a Chinese microblogging service that possesses some cultural and language differences relative to foreign SNSs. In fact, Sina Weibo can be considered a hybrid of Twitter and Facebook (Zhu, 2012) and not a Twitter clone. Sina intended to make Sina Weibo a “one-way plus two-way” relationship platform that values not only the one-way communication from followee to follower but also the two-way interaction that occurs when the two individuals (followee and follower) follow each other. Thus, this site is a unique information system (IS). To the best of our knowledge, few studies have examined the factors underlying the interests of users who continue to use it. The mechanisms that underlie the continuance intentions and behaviors of users should be explored in the context of Chinese culture. Thus, our study aims to reveal the motivations behind continued user interest, and understand and evaluate the processes through which Sina Weibo motivate continued usage. This information could aid in the elucidation of the mechanisms behind the rising usage of this site and provide insight that may contribute to the success of future enterprises.

The two main avenues of research on the use of IS are as follows: acceptance research, which primarily employs the technology acceptance model (TAM; Davis, 1989), and IS continuance research, which mainly adopts the expectation confirmation theory (ECT; Oliver, 1980). To assess the continuance intentions of Sina Weibo users, we chose the expectation confirmation model (ECM; Bhattacherjee, 2001a) in IS continuance theory as the basic theoretical framework of the research model and integrated constructs of habit (Limayem et al., 2003, 2007; Barnes and Bohringer, 2011) and perceived critical mass (Lou et al., 2000; Van Slyke et al., 2007; Barnes and Bohringer, 2011) into the model. Muscanell and Guadagno (2012) suggested that gender moderates the activities that users engage in while using SNSs. Therefore, we particularly focused on the moderating effect of gender on the continuance intention of users in our research model.

The remainder of the paper is structured as follows: a brief review of the theoretical background and our research model and hypotheses are presented in the next section. Then, we describe the methodology of our empirical study and the research results. Finally, we discuss the findings and present conclusions.

## Literature Review and Hypotheses

**FIGURE 1:**
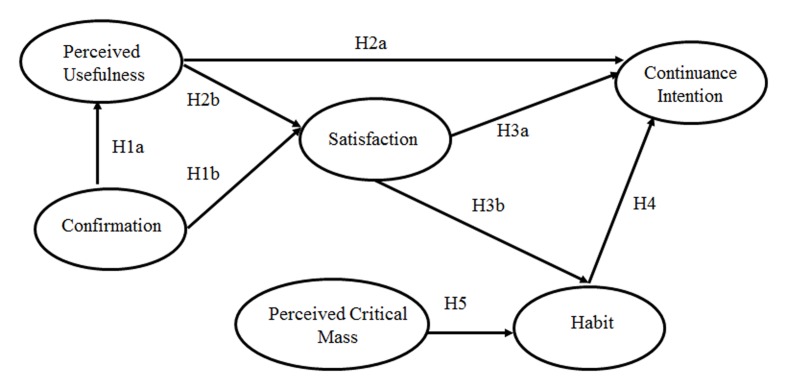
**The research model: an extended expectation confirmation model**.

In this section, we briefly review the theoretical background of our research model and present the development of our hypotheses. There are four main branches of theory that contributed to the development of this model (**Figure 1**): the ECM, which is the main framework; constructs of habit and perceived critical mass; and the gender effect. These branches are described in the sections that follow.

### Expectation Confirmation Model

Originally, Oliver (1980) proposed the ECT in the marketing field. This theory established the basic framework for evaluating the relationship between general consumer satisfaction and post-purchase behaviors. However, given the congruence between the continuance usage of an IS and the repeat purchase behaviors of consumers, Bhattacherjee (2001a) suggested using ECT to explain the continuance intention of users in relation to IS. He borrowed the concept of “perceived usefulness” from the TAM to replace “expected usage” and modified the ECT to generate the ECM. Bhattacherjee (2001a) empirically tested an ECM of e-banking service continuance and showed that the ECM could be applicable in an IS context. Since then, the ECM has been widely applied to research IS continuance. In this model, three variables, confirmation, perceived usefulness, and satisfaction, are used to explain continuance intention.

Before users adopt an IS, they must form an initial expectation of its performance. During the initial IS user experience, these individuals evaluate whether their expectations have been met. Thus, confirmation is a cognitive belief that represents the extent to which ex ante expectations are met realistically. Bhattacherjee (2001a) defined confirmation as the perceptions of users regarding the congruence between their usage expectations and the actual performance of the IS.

Based on the cognitive dissonance theory (Festinger, 1962), Bhattacherjee (2001a) explained that confirmation could promote perceived usefulness by users and that disconfirmation could reduce it. Many studies have empirically verified that confirmation is a significant predictor of perceived usefulness in the context of IS use (Bhattacherjee, 2001a; Lin et al., 2005; Barnes and Bohringer, 2011). According to the ECM, if users’ expectations regarding the performance of an IS are confirmed during actual use, the users are satisfied. If the expectation is not confirmed, users are disappointed. In other words, confirmation (i.e., actual performance is higher than initial expectations) results in high satisfaction, and disconfirmation (i.e., actual performance is lower than initial expectations) results in low satisfaction. Previous studies involving IS use have verified this confirmation–satisfaction association (Bhattacherjee, 2001a,b; Bhattacherjee and Premkumar, 2004; Hsu and Chiu, 2004; Barnes and Bohringer, 2011). In summary, the extent of confirmation by IS users positively relates to their perceptions of the usefulness of the system and their satisfaction levels. We propose the following:

H1a: The extent of confirmation by users is positively related to perceived usefulness.
H1b: The extent of confirmation by users positively influences their satisfaction levels.

Users adopt an IS with a specific goal in mind. Therefore, if they continue using the IS, they should benefit from it. According to Davis (1989), perceived usefulness refers to the degree to which users perceive that using an IS will improve their performance. Perceived usefulness is the users’ subjective perception of the expected benefits of IS use and not an objective assessment. If users consider an IS to be useful, they tend to continue using it. Based on the TAM, many prior studies (Davis, 1989; Mathieson, 1991; Taylor and Todd, 1995; Moon and Kim, 2001; Hsu and Lin, 2008) have found that perceived usefulness is related to IS acceptance intention. In the ECM, Bhattacherjee (2001a) posited that the usefulness-intention association could be applied to the IS continuance context, and this relationship was supported by empirical evidence. Therefore, the greater a user perceives the usefulness of a service to be, the higher the user’s satisfaction will be. Thus, the perceived usefulness by users is positively related to their satisfaction with the IS. We wish to demonstrate the same relationships here as follows:

H2a: Perceived usefulness positively influences Sina Weibo usage continuance intention.
H2b: Perceived usefulness is positively related to user satisfaction with Sina Weibo.

According to Kim (2012), user satisfaction is defined as “an ex-post evaluation based on user experience with a target service or product and is captured as a negative feeling, indifference, or a positive feeling by comparing the perceived performances of a service or product with their expectations” (Oliver, 1993; Spreng et al., 1996). Bhattacherjee (2001a) also provided the following operational definition of satisfaction: the feelings of users regarding their prior IS usage. In consideration of the ECM (Bhattacherjee, 2001a), when the actual performance of the IS achieves or exceeds user expectations, users will be satisfied with it. Generally, if users are satisfied with an IS, they tend to continue using it. User satisfaction is the primary determinant of IS continuance intention, and prior studies have provided empirical support for this relationship (Bhattacherjee, 2001a,b; Barnes and Bohringer, 2011; Halilovic and Cicic, 2013). Besides’ relatedness needs influence user satisfaction. A recent study shows that Facebook use helps satisfy users’ positive relatedness needs which enhances their satisfaction and motivates sustained engagement (Sheldon et al., 2011). Thus, IS user satisfaction positively influences continuance intention. Hence, we propose the following:

H3a: Satisfaction positively influences Sina Weibo usage continuance intention.

### Integrating Habit into the ECM

Limayem et al. (2007) systematically summarized prior research studies on habit and defined habit in the context of IS use as “the extent to which people tend to perform behaviors (use IS) automatically because of learning.” As Triandis (1980) suggested, there are both direct and interactive effects of habit on behavior. On the one hand, if behaviors are repeated frequently, they tend to become habitual over time (Ouellette and Wood, 1998). On the other hand, the effects of habit on actual behavior are mediated by intention (Beck and Ajzen, 1991; Quine and Rubin, 1997; Saba and Di Natale, 1998; Orbell et al., 2001), and habit moderates the relationship between intentions and actual behavior (Verplanken et al., 1997; Limayem et al., 2003). Recently, Limayem et al. (2007) found that habit significantly moderates the relationship between IS continuance intention and continued usage. However, our study focuses on intention rather than continuance usage. Thus, as Barnes and Bohringer (2011) suggested, we will focus on the direct effect of habit on continuance intention, and we hypothesize as follows:

H4 Habit positively influences the continuance intention of Sina Weibo users.

Additionally, user satisfaction is one of the key factors of habit formation. If a behavior creates satisfactory experiences for a user, then the user tends to repeat that behavior frequently. In the context of IS use, some studies have verified that satisfaction is closely related to habit development (Reibstein, 2002; Limayem et al., 2007; Barnes and Bohringer, 2011). Thus, we posit that habit is positively influenced by user satisfaction as follows:

H3b: Satisfaction has a positive influence on user habits.

### Integrating Perceived Critical Mass into the ECM

Critical mass is one important factor that influences technology adoption and the diffusion of innovation. According to Rogers (1995), critical mass refers to the threshold at which a certain minimum number of users have adopted an innovation. Exceeding this point, the rate of adoption becomes self-sustaining and creates further growth. Thus, critical mass is the basis for collective actions. Although critical mass is important, it is difficult to measure. Lou et al. (2000) proposed the perceived critical mass, which refers to the potential perceptions of new users regarding whether the innovation has attracted a critical mass of users and influences their subsequent adoption and use of the innovation, as a means of measuring critical mass. Based on the TAM, Lou et al. (2000) demonstrated that perceived critical mass could both directly and indirectly influence intentions to use groupware. However, Barnes and Bohringer (2011) posited that critical mass will indirectly affect continuance intention through automatic behaviors. In accordance with Markus (1994), the greater the perceived critical mass is, the greater the habitual behavior users will exhibit. Thus, we suggest that perceived critical mass positively influences user habits and that perceived critical mass indirectly affects the continuance intentions of users via habit, and we hypothesize the following:

H5: Perceived critical mass positively influences habit.

### Gender Effect

Gender is a fundamental sociocultural factor. As Eagly (1987) suggested, men and women have differing interests in activities; traditionally, men tend to be task-oriented, and women tend to be communally oriented. Gender differences have significant effects on the perceptions of individuals and their behaviors. Regarding IS use, particularly in the context of online settings, there are salient gender differences in users’ usage purposes for social networks. For instance, females are more likely to use social networks for relationship maintenance, and males are more likely to use them to form new relationships (Muscanell and Guadagno, 2012). Gender differences also exist in the attitudes of users toward technology (Venkatesh and Davis, 2000). Many prior studies (Venkatesh et al., 2003; Ahuja and Thatcher, 2005; Ilie et al., 2005) have demonstrated the important moderating effects of gender differences on technology adoption and use. It is reasonable to assume that there are some differences in Sina Weibo continuance intention across genders. We may achieve a better understanding of and insight into the gender differences of Sina Weibo users’ continuance intentions by examining the moderating effect of gender. Hence, we will test the moderating effect of gender in our research model.

## Materials and Methods

### Instrument Development

The questionnaire was developed to measure and verify the research model. To ensure content validity, the questionnaire items were selected from previous studies (Bhattacherjee, 2001a; Limayem et al., 2007; Van Slyke et al., 2007). The procedure of Chinese translation was as follows: first, two graduate students and an assistant professor of psychology independently translated the English version of these items into Chinese. After translation, the translators discussed their versions and generated a single draft. Second, the unanimous version was translated back into English to examine whether there were translation errors. Third, “Twitter” was replaced with “Weibo” in the questionnaire. Finally, three Weibo users were recruited to complete the questionnaire, and we modified and finalized the instrument based on their feedback. All of the items were measured using a 7-point Likert scale, where 1 = strongly disagree and 7 = strongly agree. There were 6 constructs, including perceived usefulness (PU; three items, CR = 0.871), confirmation (CONF; three items, CR = 0.852), satisfaction (SAT; three items, CR = 0.895), habit (HABIT; three items, CR = 0.841), perceived critical mass (PCM; four items, CR = 0.896), and continuance intention (CONTIN; three items, CR = 0.825). All of the constructs satisfied the validity and reliability criteria (see “Measurement Model” Section). The survey items are listed in (**Appendix Table A1**).

### Participants and Data Collection

We downloaded 100,000 user IDs by calling Sina Weibo’s open application programming interface (API) according to the following three criteria: (1) the user IDs were registered at least 1 month before the last update was posted; (2) the IDs were associated with no more than 100 status updates per day; and (3) the IDs were associated with at least one update since 2012. We randomly selected 30,000 users from the pool and invited them to take part in our online survey, which ran from May 1 to July 22 in 2012, by using the “@” function of Weibo. In total, 505 users responded, and they were paid CNY30 (approximately $4.8). Seven responses were discarded because of incomplete data, resulting in a total of 498 responses. **Table 1** shows the demographic profile of the surveyed respondents. The sample set is composed of 38.0% males and 62.0% females. Approximately 41.4% of the respondents were less than 21 years old, 38.1% were 21–25 years old, 14.5% were 26–30 years old, and only 6% were over 30 years old. Approximately 30.5% of the respondents held a high school diploma or less, 22.1% held a junior college diploma, 42.2% held a bachelor degree, and 5.2% held a graduate degree. This study was approved by the Institutional Review Board of the Institute of Psychology of the Chinese Academy of Sciences.

**Table 1:** Descriptive statistics on the respondents’ demographics.

Demographics	Frequency	Percentage
**Gender**		
Male	189	38.0
Female	309	62.0
**Education**		
High school or below	152	30.5
Junior college	110	22.1
Bachelor degree	210	42.2
Graduate degree	26	5.2
**Age**		
<21	206	41.4
21–25	190	38.1
26–30	72	14.5
>30	30	6.0


### Data Analyses

To analyze the data, we used the SmartPLS 2.0 software (Ringle et al., 2005), which is a partial least squares (PLS)-based tool. This research uses PLS for two reasons. First, PLS is a non-parametric procedure that is suitable when data are non-normally distributed (Falk and Miller, 1992; Qureshi and Compeau, 2009). Second, PLS is appropriate for the current research because it is preferable for prediction purposes (Fornell and Bookstein, 1982). We performed a confirmatory factor analysis (CFA) to assess the measurement model and examined the structural model with a structural equation modeling (SEM) approach. First, we used the entire sample to examine the baseline model. Then, we performed a multi-group analysis of the male and female samples to examine the gender effect.

## Results

### Measurement Model

We first tested the validity and reliability of the measurements, for which convergent validity and discriminant validity were verified. As shown in **Table 2**, the questionnaire items had high levels of convergent validity, and the item loadings ranged from 0.598 and 0.886 and were all higher than 0.55 (Tabachnick and Fidell, 2007). The higher the loading value, the more accurate the item is as a measurement of the construct. As Comrey and Lee (1992) suggested, a loading above 0.55 (30% overlapping variance) is acceptable for interpreting the construct.

**Table 2:** Psychometric index of measurements.

Constructs	Items	Loading	Standard error	CR	AVE
Perceived usefulness	PU1	0.827	0.034	0.871	0.692
	PU2	0.850	0.026		
	PU3	0.818	0.031		
Confirmation	CONF1	0.748	0.047	0.852	0.658
	CONF2	0.820	0.029		
	CONF3	0.862	0.019		
Satisfaction	SAT1	0.857	0.024	0.895	0.740
	SAT2	0.837	0.028		
	SAT3	0.886	0.019		
Habit	HABIT1	0.848	0.024	0.841	0.639
	HABIT2	0.730	0.038		
	HABIT3	0.814	0.035		
Perceived critical mass	PCM1	0.811	0.029	0.896	0.683
	PCM2	0.785	0.043		
	PCM3	0.860	0.024		
	PCM4	0.848	0.028		
Continuance intention	CONTIN1	0.869	0.024	0.825	0.617
	CONTIN2	0.598	0.087		
	CONTIN3	0.859	0.022		


To test the reliability, we calculated the composite reliability (CR) and average variance extracted (AVE). Fornell and Larcker (1981) suggested that if the CR value is above 0.70 and the AVE value is above 0.50, then the reliability is acceptable. **Table 2** demonstrates that the reliability of all of the corresponding constructs was higher than the recommended level.

We used the procedures proposed by Fornell and Larcker (1981) to test the discriminant validity. In these procedures, if the square root of the AVE for each construct is higher than its correlations with the other constructs, then the discriminant validity is verified. As shown in **Table 3**, all of the square roots of the AVE for each construct were higher than the correlations. Diagonal elements are the square roots of the AVE for the corresponding construct.

**Table 3:** Correlations between constructs.

	PU	CONF	SAT	HABIT	PCM	CONTIN
Perceived usefulness (PU)	0.832					
Confirmation (CONF)	0.811	0.821				
Satisfaction (SAT)	0.723	0.684	0.860			
Habit (HABIT)	0.755	0.735	0.672	0.799		
Perceived critical mass (PCM)	0.567	0.601	0.512	0.635	0.826	
Continuance intention (CONTIN)	0.743	0.710	0.686	0.755	0.539	0.785


Overall, in terms of the psychometrics, the validity and reliability of the questionnaire were acceptable. Thus, we were able to use the data to estimate the structural model and test the hypothesis.

### Structural Model and Hypothesis Testing

#### Results of Structural Model for Whole Sample Set

**FIGURE 2:**
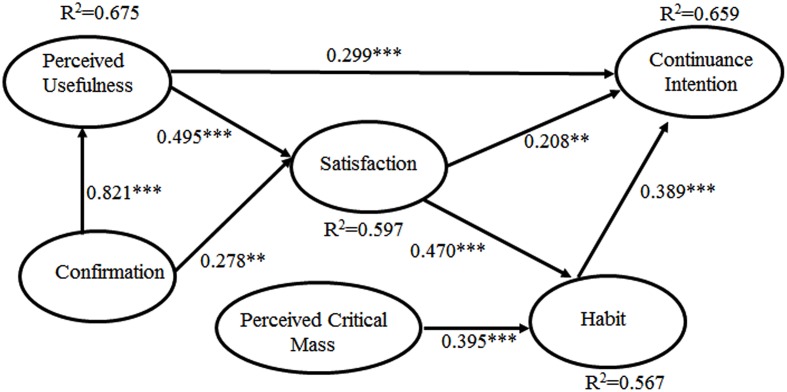
**Sina Weibo continuance intention for the whole sample set.** ^∗^*P* < 0.05; ^∗∗^*P* < 0.01; ^∗∗∗^*P* < 0.001.

We used the whole sample set to assess the structural model and test the research hypotheses. The structural model results are shown in **Figure 2**, and **Table 4** depicts the direct and indirect effects of the variables on the intention to continue using Sina Weibo. The users’ intentions to continue using the system were directly predicted by perceived usefulness (β = 0.299), satisfaction (β = 0.208), and habit (β = 0.389), which jointly explained 65.9% of the variance in intention. In addition to the direct effects, perceived usefulness and satisfaction also have indirect effects on the Sina Weibo users’ intentions to continue using the system. As shown in **Table 4**, the indirect effects of perceived usefulness on continuance intention were 0.194, and the total effect was 0.493. The indirect effect of satisfaction on the continuance intention was 0.183, and the total effect was 0.391. All of these effects were significant. Therefore, H2a (PU-CONTIN), H3a (SAT-CONTIN), and H4 (HABIT-CONTIN) are supported.

**Table 4:** Effects on Sina Weibo usage continuance intention.

Construct	Direct Effects	Indirect Effects	Total Effects
Perceived usefulness	0.299	–	0.493
		0.103	
		0.091	
Confirmation	–	–	0.512
		0.245	
		0.085	
		0.058	
		0.051	
		0.074	
Satisfaction	0.208	–	0.391
		0.183	
Habit	0.389	–	0.389
Perceived critical mass	–	–	0.154
		0.154	


Satisfaction is influenced directly and positively by confirmation (β = 0.278) and perceived usefulness (β = 0.495), which together explain 54.8% of the variance in satisfaction, and H1b (CONF-SAT) and H2b (PU-SAT) are supported.

Satisfaction (β = 0.0.470) and perceived critical mass (β = 0.395) have a direct and positive influence on habit, and 56.7% of the variance in habit is explained by satisfaction and perceived critical mass. Hence, H3b (SAT-HABIT) and H5 (PCM-HABIT) are also supported.

Confirmation can positively predict perceived usefulness (β = 0.821) and explains 67.5% of the variance in perceived usefulness, thus supporting H1a (CONF-PU).

#### Moderating Effect of Gender

In addition to the effects of the aforementioned predictors on the continuance intention, we were also concerned with the moderating effect of gender. The results of the multi-group analysis of gender are presented in **Table 5.** Overall, all of the research hypotheses are supported by both the male and female data samples. However, there were two differences in the path coefficients by gender, demonstrating the moderating effect of gender on the model. For H4, the path coefficient from habit to continuance intention for males (β = 0.268) was significantly smaller than that for females (β = 0.485). In contrast, the result of H2a showed the opposite. The path coefficient from perceived usefulness to continuance intention for males (β = 0.414) was marginally significantly larger than that for females (β = 0.228). Therefore, habit plays a more important role for continuance intention in females than in males, but perceived usefulness seems to be more important for males than for females.

**Table 5:** Moderating effect of gender.

Hypotheses	Male		Female		*T*-value (difference between genders)
			
	Path coefficients	*SE*	Path coefficients	*SE*	
H1a (CONF-PU)	0.843^∗∗∗^	0.022	0.805^∗∗∗^	0.025	1.049
H1b (CONF-SAT)	0.271^∗∗^	0.080	0.272^∗∗^	0.091	0.008
H2a (PU-CONTIN)	0.414^∗∗∗^	0.074	0.228^∗∗^	0.074	1.680^†^
H2b (PU-SAT)	0.530^∗∗∗^	0.086	0.471^∗∗∗^	0.110	0.379
H3a (SAT-CONTIN)	0.210^∗∗^	0.066	0.219^∗∗^	0.064	0.093
H3b (SAT-HABIT)	0.528^∗∗∗^	0.058	0.412^∗∗∗^	0.073	1.120
H4 (HABIT-CONTIN)	0.268^∗∗^	0.077	0.485^∗∗∗^	0.062	2.184^∗^
H5 (PCM-HABIT)	0.394^∗∗∗^	0.056	0.399^∗∗∗^	0.069	0.051


*^†^p < 0.01; ^∗^p < 0.05; ^∗∗^p < 0.01; ^∗∗∗^p < 0.001.*

## Discussion

The aim of this research was to examine the factors influencing users’ intentions to continue using Sina Weibo. The data collected from users were utilized to test the proposed research model. To validate the research model for Sina Weibo users’ continuance intentions, SEM was carried out, and a multiple-group comparison was performed to examine the moderating effect of gender. The results showed that the users’ continuance intentions were determined by their perceptions of the usefulness of the system and their satisfaction and habits, and the proposed extended ECM explained 65.9% of the total variance in the users’ continuance intentions. In addition, there were some gender differences in continuance intentions.

### Understanding the Relationship between Determinants and Continuance Intention

From the IS continuance perspective, we have examined the effects of perceived usefulness, confirmation, satisfaction, habit, and perceived critical mass on the continuance intentions of Sina Weibo users via the proposed extended ECM. The results of this research demonstrate that perceived usefulness, satisfaction, and habit are significant determinants of users’ continuance intentions.

In line with expectations, satisfaction and perceived usefulness were shown to be significant predictors of continuance intention. This finding is consistent with the findings of prior ECM-based studies (Bhattacherjee, 2001a; Barnes and Bohringer, 2011; Halilovic and Cicic, 2013). However, in this study, the effect of perceived usefulness on users’ continuance intentions was larger than that of satisfaction, which differs from what was observed by Bhattacherjee (2001a), who reported that satisfaction with IS use was a stronger predictor of continuance intention than perceived usefulness. The present results are in agreement with those of prior TAM-based studies of IS acceptance indicating that perceived usefulness is a stronger predictor of acceptance intention in TAM than attitude (Davis, 1989; Taylor and Todd, 1995). Furthermore, in previous ECM-based studies (Barnes and Bohringer, 2011), perceived usefulness was a stronger predictor of users’ continuance intentions than satisfaction. In general, perceived usefulness is a cognitive belief, but attitude and satisfaction reflect users’ feelings (pre- and post-acceptance). Although the predictive power of these two factors (perceived usefulness and satisfaction) varies across contexts, the associations between them and IS acceptance and continuance intention are robust and salient.

Furthermore, Sina Weibo users’ habits had significant positive effects on their continuance intentions. These findings are consistent with previous work studying IS continuance (Limayem et al., 2007; Barnes and Bohringer, 2011; Venkatesh et al., 2012). This result can be interpreted as showing that if users have formed a habit of using Weibo (i.e., usage behaviors become automatic), they tend to continue to use it.

### Understanding the Relationship between Determinants

In this research, the results showed that perceived usefulness has a stronger effect on satisfaction than confirmation, indicating that users’ perceptions of usefulness are key determinants of their satisfaction levels. Furthermore, confirmation is positively related to perceived usefulness and satisfaction. Thus, there are two indirect ways that confirmation influences continuance intention: via perceived usefulness and via user satisfaction. These findings are in accordance with previous ECM-based studies (Bhattacherjee, 2001a; Barnes and Bohringer, 2011; Halilovic and Cicic, 2013).

In addition, this study found that satisfaction and perceived critical mass are two primary antecedents to habit development. As Aarts et al. (1997) suggested, because a satisfactory experience with a behavior increases one’s tendency to repeat the same course of action, satisfaction is a key condition for habit development. Regarding IS continuance, some recent studies have found that satisfaction positively influences user habits (Barnes and Bohringer, 2011). According to Markus (1994), the greater the perceived critical mass is, the greater the habitual behavior that users will exhibit. Barnes and Bohringer (2011) also found that perceived critical mass positively influences user habits. Hence, user satisfaction and perceived critical mass contribute to the development of user habits.

### Understanding the Moderating Effect of Gender

The multiple-group comparison revealed that gender differences influence Sina Weibo users’ continuance intentions. For example, although perceived usefulness significantly affects both male and female user intentions, the role of perceived usefulness is more important for explaining male user intentions than those of females. Thus, men appear to have greater intentions to continue using Sina Weibo when they believe that it is useful. These results are consistent with previous studies (Venkatesh and Morris, 2000; Venkatesh et al., 2003; Ong and Lai, 2006; Hanudin, 2007) reporting similar findings. Males are considered to be task-oriented (Eagly, 1987), and to achieve goals, they will take the factor of usefulness into consideration to a greater degree than will females.

In contrast, habit plays a more important role in predicting female Sina Weibo users’ continuance intentions compared with males. Among the three direct predictors—perceived usefulness, satisfaction, and habit—habit had the strongest effect on female users’ continuance intention, but perceived usefulness was the strongest predictor of the continuance intentions of males. This result implies that the development of automatic behavior is more important for females to continue using Sina Weibo and that efficiency and effectiveness are more salient for males. These findings are related to the gender differences that have been reported in previous studies of IS and technology use (Hargittai, 2007; Raacke and Bonds-Raacke, 2008; Guadagno et al., 2011; Mazman and Usluel, 2011; Muscanell and Guadagno, 2012). For instance, females have been reported to be more likely to use an IS to engage in social interactions (e.g., maintaining relationships), and males tend to focus more on task-oriented activities (e.g., obtaining information and learning about news events; Eagly, 1987; Weiser, 2000, 2001). Muscanell and Guadagno (2012) also found that females used SNSs for relationship maintenance. Hence, once females have developed a habit of using Sina Weibo to interact with their friends and family, they will continue using it. Thus, habit formation plays an important role in driving females to continue using this site. Based on the current results, gender moderates the effects of perceived usefulness and habit on Sina Weibo users’ continuance intentions. Interestingly, the results of this study show that there is no difference in the effect of satisfaction on user intentions. Although men and women differ in their reasons for using Weibo, perhaps their needs are equally met by this site, and they are both satisfied with it.

## Conclusion

This study incorporates constructs of habit and perceived critical mass into the framework of the ECM to develop a research model of Weibo users’ continuance intentions and examines the moderating effect of gender. The research model is empirically verified using an online survey of 498 Weibo users. Perceived usefulness, satisfaction, and habit are significant determinants of users’ continuance intentions. Perceived usefulness has a strong effect on satisfaction, and confirmation is positively related to perceived usefulness and satisfaction. In turn, satisfaction and perceived critical mass are two primary antecedents to habit development. Moreover, there is a gender difference in the relationship between habit and Sina Weibo users’ continuance intentions—habit plays a more important role in continuance intention for females than for males.

## Author Contributions

Conceived and designed the experiments: WQ and XS. Performed the experiments: LW, WQ, and XS. Analyzed the data: LW and RZ. Drafted the manuscript: LW. Revised manuscript: RZ and WZ.

## Conflict of Interest Statement

The authors declare that the research was conducted in the absence of any commercial or financial relationships that could be construed as a potential conflict of interest.

## APPENDIX

**Table A1:** **Questionnaire items**.

Constructs	Item
Confirmation	CONF1: My experience with using Sina Weibo was better than what I expected.
	CONF2: The service level provided by Sina Weibo was better than what I expected.
	CONF3: Overall, most of my expectations about using Sina Weibo were confirmed.
Perceived Usefulness	PU1: Sina Weibo is beneficial for me.
	PU2: The advantages of Sina Weibo outweigh the disadvantages.
	PU3: Overall, using Sina Weibo is advantageous.
Satisfaction	How do you feel about your overall experience of Sina Weibo use:
	SAT1: Very displeased/Very pleased
	SAT2: Very unconducive/Very conducive.
	SAT3: Very uninterested/Very interested.
Habit	Habit1: Using Sina Weibo has become automatic to me.
	Habit2: Using Sina Weibo is natural to me.
	Habit3: When faced with a particular task, using Sina Weibo is an obvious choice for me.
Perceived critical mass	PCM1: Many people I communicate with use Sina Weibo.
	PCM2: The people I communicate with will continue to use Sina Weibo in the future.
	PCM3: The people who I communicate with using Sina Weibo will continue to use Sina Weibo in the future.
	PCM4: Of the people who I communicate with regularly, many use Sina Weibo.
IS continuance intention	CONTIN1: I intend to continue using Sina Weibo rather than discontinue using it.
	CONTIN2: My intentions are to continue using Sina Weibo rather than using any alternative.
	CONTIN3: If I could, I would like to discontinue my use of Sina Weibo.
